# Starved? Time for SNAC1: A nitrogen starvation–responsive transcription factor that promotes nitrate uptake

**DOI:** 10.1093/plphys/kiad308

**Published:** 2023-05-24

**Authors:** Alaeddine Safi

**Affiliations:** Assistant Features Editor, Plant Physiology, American Society of Plant Biologists, USA; Department of Plant Biotechnology and Bioinformatics, Ghent University, B-9052 Ghent, Belgium; VIB Center for Plant Systems Biology, B-9052 Ghent, Belgium

Nitrogen (N) is a major limiting nutrient for plant survival and thus for food security. One of the characteristics of modern agriculture is the intensive application of chemical fertilizers as a way to secure crop yields. Although this strategy helped to cope with the N shortage in farmlands, it simultaneously came with tremendous economic and environmental repercussions. Not only is the industrial production of fertilizers extremely energy intensive, but also a substantial fraction of fertilizers, when applied, is lost in drainage water or degraded into nitrous oxide, a very potent greenhouse gas. In short, overfertilization can promote aquatic ecosystem eutrophication, accelerate soil degradation, and contribute to global warming (Sutton et al. 2011). It is of paramount importance, therefore, that fertilizer use is rationalized and also that we improve plant N use efficiency (NUE), which is as yet notoriously poor in crops.

NUE is mainly governed by N transport and assimilation rates. In the case of nitrate, its transport is led by NITRATE TRANSPORTERs (NRTs). Nitrate is subsequently reduced into ammonium and incorporated into amino acids through the sequential actions of different enzymes. Because the expression of these transporters and enzymes is tightly regulated by numerous transcription factors (TFs), many studies focused on the investigation of those TFs that fine-tune this transcriptional control (Alvarez et al. 2019, 2020; Safi et al. 2021).

In this issue of *Plant Physiology*, Qi et al. (2023) unraveled a new function of the STRESS-RESPONSIVE NAC 1 (OsSNAC1) TF in regulating nitrate utilization in rice and proposed potential prospects to improve plant growth and yield under different N conditions. OsSNAC1 was previously reported to confer tolerance to salt, drought, and oxidative stress in rice (Hu et al. 2006; You et al. 2014). In this study, OsSNAC1 recurrently emerged in a yeast 1-hybrid screen with the high-affinity nitrate transporter *OsNRT2.1* promoter, suggesting that OsSNAC1 could act as a transcriptional regulator of *OsNRT2.1* (Qi et al. 2023). This observation was further corroborated by additional approaches. Indeed, using a targeted yeast 1-hybrid, transactivation assay, and chromatin immunoprecipitation followed by quantitative PCR, OsSNAC1 was found to bind and activate several promoter regions of *OsNRT2.1* and other nitrate transporters (*OsNRT2.2*, *OsNRT1.1A*, and *OsNRT1.1B*). Interestingly, *OsSNAC1*, *OsNRT2.1*, and *OsNRT2.2* response to nitrate is reminiscent of the expression pattern of the N starvation markers *AtNRT2.4* and *AtNRT2.5* (Safi et al. 2021). Not only are all of these genes induced by N deficiency, but they also display a transient induction within the first 30 min of nitrate treatment, followed by a decline in the subsequent hours (Safi et al. 2021).

To dig into the role of OsSNAC1 in N nutrition, the authors utilized 2 independent overexpressor lines (OsSNAC1_OE) and 2 independent loss of function mutants (*Ossnac1*). Consistent with the transactivation assay, the aforementioned nitrate transporters were upregulated in the OsSNAC1_OE lines. As expected, the increase in gene expression of the *NRT*s in these plants correlated with higher nitrate uptake and NUE rates. Moreover, overexpressing *OsSNAC1* also promoted plant height and biomass, root length, and grain yield. By contrast, *Ossnac1* mutants were negatively affected in *NRT* gene expression, nitrate uptake and NUE, and plant growth and yield (Qi et al. 2023).

In summary, Qi et al. (2023) present a novel role of OsSNAC1 and propose a new TF-*NRT* module with functional consequences on N acquisition and plant growth. A number of NAC TFs in different plant species are known to be involved in analogous pathways (He et al. 2015; Alvarez et al. 2019; Tang et al. 2019). For example, similar to *OsSNAC1*, *OsNAC42* is also responsive to N deficiency and can regulate NUE and plant growth via inducing *NRT* genes (Tang et al. 2019). Thus, it would be of interest to examine a potential functional redundancy between those 2 genes.

Because *NRT* promoters are tightly controlled by multiple TFs, it would be interesting to integrate their combinatory effects. For instance, combining the manipulation of the repressive TFs (e.g. HRS1 homologs) (Safi et al. 2017) with the activator ones (e.g. OsSNAC1, OsNAC42) might notably intensify the desirable phenotypes under low-N conditions. Alternatively, a synthetic biology approach could be envisaged to meticulously eliminate the repressible cis-regulatory elements of (*CREs*) or cluster the inducible *CREs* within *NRT* promoters. Finally, because OsSNAC1 is also involved in other abiotic stress responses such as salt and drought tolerance (Hu et al. 2006; You et al. 2014), one might consider exploring its role in the context of combinatorial stress conditions (e.g. N starvation + drought).
